# Euthanasia requests in dementia cases; what are experiences and needs of Dutch physicians? A qualitative interview study

**DOI:** 10.1186/s12910-019-0401-y

**Published:** 2019-10-04

**Authors:** Jaap Schuurmans, Romy Bouwmeester, Lamar Crombach, Tessa van Rijssel, Lizzy Wingens, Kristina Georgieva, Nadine O’Shea, Stephanie Vos, Bram Tilburgs, Yvonne Engels

**Affiliations:** 1 General practice Ottenhoff, B. Ottenhoffstraat 18, 6561 CM Groesbeek, The Netherlands; 2 Radboud University Honours Academy, Houtlaan 4, 6525 XZ Nijmegen, The Netherlands; 3 Radboud university medical center, Postbox 9101, 6500 HB Nijmegen, The Netherlands

**Keywords:** Euthanasia, Dementia, Elderly, Elderly care physician general practitioners, Primary care, Support

## Abstract

**Background:**

In the Netherlands, in 2002, euthanasia became a legitimate medical act, only allowed when the due care criteria and procedural requirements are met. Legally, an Advanced Euthanasia Directive (AED) can replace direct communication if a patient can no longer express his own wishes. In the past decade, an exponential number of persons with dementia (PWDs) share a euthanasia request with their physician. The impact this on physicians, and the consequent support needs, remained unknown. Our objective was to gain more insight into the experiences and needs of Dutch general practitioners and elderly care physicians when handling a euthanasia request from a person with dementia (PWD).

**Methods:**

We performed a qualitative interview study. Participants were recruited via purposive sampling. The interviews were transcribed verbatim, and analyzed using the conventional thematic content analysis.

**Results:**

Eleven general practitioners (GPs) and elderly care physicians with a variety of experience and different attitudes towards euthanasia for PWD were included. Euthanasia requests appeared to have a major impact on physicians. Difficulties they experienced were related to timing, workload, pressure from and expectations of relatives, society’s negative view of dementia in combination with the ‘right to die’ view, the interpretation of the law and AEDs, ethical considerations, and communication with PWD and relatives. To deal with these difficulties, participants need support from colleagues and other professionals. Although elderly care physicians appreciated moral deliberation and support by chaplains, this was hardly mentioned by GPs.

**Conclusions:**

Euthanasia requests in dementia seem to place an ethically and emotionally heavy burden on Dutch GPs and elderly care physicians. The awareness of, and access to, existing and new support mechanisms needs further exploration.

## Background

In the Netherlands, in 2002, euthanasia became a legitimate medical act, allowed only when the due care criteria and procedural requirements are met [1]. Being faced with a euthanasia request can have a major impact on physicians [2, 3]; 25% of the physicians who received such a request, experienced problems during the decision making process [4]. These problems mainly concerned the evaluation of the due care criteria. A survey showed that nearly one-third of all physicians experienced pressure from the patient (29%) or from the family (34%) during this process. Moreover, more than half of the general practitioners (GPs; 56%) and 40% of the elderly care physicians experience pressure to hasten the procedure [5]. This is related to changing views in Dutch society in the direction of being in control of your own life and a right to die [6].

More than a decade ago, several studies focused on the debate on euthanasia and on advance euthanasia directives (AEDs) for patients with dementia (PWD) [7, 8], and on experiences of physicians in nursing homes on this topic [9]. At that time, elderly care physicians and relatives appeared to be reluctant to adhere to AEDs. However, since then a lot has changed in the public debate, the opinion of physicians and in daily practice. In 2012, 40% of the physicians found it conceivable that they would grant a euthanasia request of a person with early-stage dementia, and 29–33% in persons with advanced dementia [10]. Moreover, in 2010, euthanasia for PWD took place in 25 cases (of 3136 cases in total), in 2014 in 81 cases (of 5306 in total), and in 2018 in 146 cases (of 6126 in total), mostly in competent PWD [11, 12]. The numbers of AEDs and euthanasia requests from PWD are larger, as not every request or AED ends up in euthanasia [12–14].

Assessing the due care criteria and the timing when it concerns a PWD is difficult [4, 10]. Therefore, until 2015, the Royal Dutch Medical Association (KNMG) directed that it was necessary that the patient verbally or non-verbally confirmed his or her actual death wish when receiving euthanasia. Though, in 2015 the KNMG published its latest guideline in which this was no longer required [15]. Although the number of countries where euthanasia and physician-assisted suicide is legalised is increasing [16], legally replacing verbal communication by an AED if a patient can no longer express his own wishes is only possible in the Netherlands.

In the past few years, three cases were reported in which patients with advanced dementia received euthanasia on the basis of an AED [11]. This led to several campaigns by physicians who opposed AED authorisation of euthanasia in case of advanced dementia due to their ethical concerns [17, 18]. Recently, a first judicial criminal investigation was opened on a physician’s actions regarding a euthanasia case in a person with advanced dementia [18].

We hypothesised that dealing with euthanasia requests from PWD, whether or not they are still competent, impacts physicians. As it is mainly general practitioners (GPs) and elderly care physicians who receive euthanasia requests from PWD [11], we aimed to get more insights in the impact of discussing euthanasia requests by PWD on these physicians by answering the following research question: what are the experiences and needs of Dutch general practitioners and elderly care physicians when handling a euthanasia request from a person with dementia?

## Methods

### Design

We performed a qualitative interview study with a direct content analysis approach. The Consolidated Criteria for Reporting Qualitative Studies (COREQ) checklist was followed to present the findings [19]. Interviews were performed between January and April 2018, at a place chosen by the interviewee. The study protocol was approved by the Radboudumc medical ethical committee (*NL2017–3862*).

### Participants and recruitment

Between December 2017 and February 2018 participants were recruited. In order to find physicians with different attitudes towards euthanasia for PWD, we purposively recruited physicians with a variety of experience in granting euthanasia for this patient group, male and female doctors, GPs and elderly care physicians. We used two different sources for recruitment. One was a list of doctors who signed a critical statement published in Dutch national newspapers about euthanasia procedures for persons with advanced dementia, and who had provided their email addresses [17, 20]. This critical statement opposed euthanasia in non-competent persons with advanced dementia. Taking account of the above-mentioned criteria, physicians from this list were invited by e-mail to take part.

The other source was the End of Life clinic. This clinic deals with euthanasia requests, and when due care criteria are met, offers euthanasia or assisted suicide to people whose own physicians are not able to, or do not want to, perform it [21]. The physicians of the End of Life clinic are less reluctant to perform euthanasia in case of PWD [22]. Via the director, two physicians employed at the End of Life clinic agreed to participate. We also used snowballing. After we reached saturation, we stopped inviting physicians to take part.All participants signed an informed consent before the interview took place.

### Data collection

Based on scientific and grey literature, the public debate and two pilot interviews which were not included in the analysis, a topic guide with four open questions was developed. (Table 1) All authors were trained in qualitative interviewing and analysis. There were two core interviewers (LC and LW); one of them was always leading the interview. During each interview, an additional researcher was present (RB, TR, LC, LW), who took notes and could ask additional questions. Anonymity of data processing was guaranteed and each participant was assured that the published results would preclude any identification of physician or patients.

**Table 1:** The Topic Guide Key Items

Topics	
1.General opinion of and experience with euthanasia requests and procedures	
2. Determinants for deciding whether euthanasia should be performed	
3 Experience with euthanasia requests and procedures in dementia cases in particular	
4. Effects on work	
5. Need for support mechanisms	
6. Suggestions	

### Data analysis

Interviews were audio-recorded, and within a few days transcribed verbatim. After two interviews, we applied conventional thematic content analysis of the transcripts to develop a codebook [23]. Two researchers independently coded each interview line-by-line, after which they discussed differences until consensus was reached. If no consensus was reached, the codes were discussed with the project leaders (YE and JS). Finally, during a meeting with all researchers, codes were merged into categories and themes, and an affinity diagram was made [24, 25]. During the coding and merging processes, quotes that best reflected the codes within a category were selected. Each quote received a letter (A to K), representing the physician who mentioned it.

## Results

After ten interviews no new codes were revealed. To confirm data saturation, one additional interview was conducted; in total eleven physicians took part. Ten of them had signed the critical statement about euthanasia in non-competent PWD [17]. Two physicians were employed at the End of Life clinic [21]. Characteristics and background of the eleven interviewees are provided in Table 2. Each interview took between 40 and 90 min.

**Table 2:** Characteristics and Participants Background (*n* = 11)

PARTICIPANTS		No. (%)
Gender		
Male		7 (64)
Female		4 (36)
Age		
40–50		3 (27)
50–60		3 (27)
60–70		2 (12)
70–80		3 (27)
Current workplace^a^		
General practice		2 (18)
End of life clinic		2 (18)
Nursing home		2 (18)
Hospital		1 (11)
Retired		2 (18)
Hospice		1 (11)
Palliative consulting team		1 (11)
Elderly care trainer		1 (11)
Participants’ basic training		
Elderly care physician		6 (55)
General practitioner		5 (45)
Participants’ experience		
*Total of euthanasia procedures*		2 (18)
0		2 (18)
1		2 (18)
2		2 (18)
3		1 (11)
> 100		2 (18)
Unknown		
*Euthanasia in dementia cases*		4 (36)
Requests	Procedures	1 (11)
0	0	1 (11)
1	0	2 (18)
2	1	1 (11)
2	0	1 (11)
3	0	1 (11)
10	6	> 40 (11) 1(11)
Unknown	> 40	1 (11)

*SCEN, ‘support and consultation during euthanasia procedure’*

^a^Multiple categories per participant possible

In all cases consensus about the coding was reached. The final code book consisted of 45 codes, which were merged into five themes: evaluation of the euthanasia request, difficulties experienced by doctors, alternatives for euthanasia, expertise, support & coping, and doctors’ emotions. (Table 3)

**Table 3:** Overview of themes, categories and codes

THEMES	CATEGORIES	CODES
Evaluation of the euthanasia request	*Reasons for rejection*	⋅ too late in the dementia trajectory
		⋅ no repeated clear request
		⋅ mental incompetence
	*Reasons for acceptance*	⋅ unbearable suffering in future
		⋅ has to feel right
		⋅ repeated clear convincing request
Difficulties experienced by doctors	*Timing*	⋅ different timing and agenda’s of doctors and patients
		⋅ diagnosis takes too long
	*Workload*	⋅ work pressure
		⋅ long preparation
		⋅ labor-intensive
	*Pressure by relatives*	⋅ pressure by family
		⋅ request from family
		⋅ part of the suffering lies with the family
	*Influence from society*	⋅ society not dementia-friendly
		⋅ euthanasia is considered a good death
		⋅ negative perspective on dementia
		⋅ slippery slope regarding granting euthanasia
		⋅ changed perspective on death and dying
		⋅ autonomy is leading
	*Patient-doctor communication*	⋅ difficult communication due to dementia
		⋅ conversation with or without family
	*Law, due care criteria and the guidelines*	⋅ unbearable suffering is unclear
		⋅ judging mental competence difficult
		⋅ vague guidelines
		⋅ AED not useful in dementia cases
		⋅ AED are complicated
Expertise	*Individual (GPs + elderly care physicians)*	⋅ improves quality on care
		⋅ experiences reduces fear
		⋅ infrequency
	*Organizational (SCEN and end-of-life clinic)*	⋅ pros: more time for patients, safety net, legal support
		⋅ cons: stigmatization, contributes to slippery slope, no negative view on euthanasia
Support and coping	*Improvement of existing conditions*	⋅ colleagues and other professionals
		⋅ buddy system
		⋅ emotional support by own family
		⋅ too costly to implement
	*Alternatives to euthanasia*	⋅ assisted suicide
		⋅ palliative care (palliative sedation)
Doctor’s emotions	*Negative*	- nervous
		⋅ frustrated
		⋅angriness
		⋅ restless·
	*Positive*	⋅ relief and satisfaction
		⋅ feeling of control
		⋅ heroism

### Evaluation of the euthanasia request

Nine of the eleven interviewees stated they would be willing to ***accept*** a euthanasia request of a PWD when he or she would still be competent and they would be convinced that the patient has, or will have unbearable, refractory existential suffering in the near future. One of the participants pointed out *‘people suffer most from the suffering that is yet to come (A)’*.

Most participants mentioned that they would ***reject*** a request or AED from a patient with an advanced stage of dementia, as they consider these patients lack mental capacity. They felt strong moral objections to performing euthanasia if they could no longer communicate effectively with their patient. ‘*Patients expect they can give you their filled-out form and that’s it. They think that it [an AED] is enough to receive euthanasia at the moment they want it’(D).* Another participant stated ‘*I have recently rejected euthanasia requests from four people. Each of these persons no longer understood what we were talking about’(C)*

### Difficulties experienced by doctors

#### Timing

Most participants said that physicians often postpone disclosure of the dementia diagnosis because of the difficulties surrounding the diagnosis process itself, but also because of the related difficulties which arise once the diagnosis is given. “*There is a conspiracy against this diagnosis, also amongst physicians. It’s Alzheimer, isn’t it? Is it D …*? *And to avoid the term they will say you’ve got a mild cognitive impairment …*.” *(C)* Postponing this disclosure conflicts with needing considerable time to consider euthanasia in a PWD, as stated by several participants. Moreover, doctors mentioned that they often have different agendas and timing regarding the actual moment of euthanasia compared to the expectations of the patients and their family. *“I think as a doctor you need to prepare this really well, not just at that particular moment, but you need to start years before. You need to discuss things and document them repeatedly.”(D).*

Particularly in dementia cases, it is difficult for the patient and for the physician to predict unbearable suffering in the future. One of the elderly care physicians said “*I find this a very difficult and complex matter, … .if they ask for death, well.., in the early stages of dementia people will say; What a waste; you’ve still got some good years ahead of you”. But if they’re late, they will say: “Well, it’s too late now, he’s gone completely nuts.” And to plan this. “That’s just as difficult as everyone thinks it is, you see.”(C)*

#### Workload

All participants agreed that spending sufficient time on the request, on consultations for comprehensive consideration, and on the actual euthanasia procedure improves the quality of care. Consequently, as stated by some participants, being confronted with euthanasia requests and procedures has a negative impact on the overall quality of care and time left for other patients. The majority of physicians underlined the time investment needed around euthanasia requests as challenging: “*genuine, unprejudiced attention … that takes time …*. *Yeah, because it interferes with daily affairs, you need to plan extra time for it, I often do this on my day off, … when I cannot be disturbed by other patients.”(G)*

#### Pressure by relatives

According to most participants, euthanasia requests often come from the family and not from the patient. Many participants had experienced situations in which family members said their parent’s life was not dignified anymore: *“Children in particular feel they are their mother’s spokesperson and will continue knocking on the GP’s door … There you have to ...there a line needs to be drawn. I get very few requests from the patients suffering from dementia themselves. It is more often a family member who says “father wouldn’t have wished this”. So a real question from the patient him- or herself, hardly ever. ...It frequently happens that family members put pressure: “Is this necessary?” and “Please administer extra syringes to make it happen more quickly” I think GPs need to be protected against this kind of pressure when the patient is suffering from dementia and does not ask for it himself.”(K)*

#### Influence from society

According to some participants, society considers dementia as a disease with hardly any quality of life, with euthanasia often being perceived as a more dignified alternative. One participant stated: “*The focus in the Netherlands is very much on euthanasia, whereas this is something to be discussed: How do we as a society deal with dementia, because most people suffering from dementia live at home and continue to do so for quite a while. Are we as a society friendly towards them and how well is their care and support organised? Not much has been arranged, so in a way they and their family caregivers have to face things alone.”(A)* Some participants argued that euthanasia is a dignified and accepted death and that the difficulty lies within our society’s emphasis on autonomy: “*Because our primary value has become taking autonomous decisions. And what happens with loss of cognition is that the autonomy is disintegrating.”(D).*

Others argued that viewing euthanasia as a synonym for dignified dying, raises questions about the dignity of our society as a whole. “*In my opinion we should not just be a dementia-friendly society, but a society which treats the elderly with more respect. A society which does not estimate people’s value by their economic contributions and by whether people still go on holidays or those kind of things”(E)*

#### Patient-doctor communication

Nearly all participants expressed difficulties in communication with PWD. They stated that it is often not clear whether a previously expressed wish to die is still really this patient’s actual desire, especially if a patient is not able to repeat his request anymore. Even if the patient has an AED it is difficult to check if this request is still valid. “*‘If I become demented and do not recognize my family anymore I want euthanasia’. And if such a person at a later moment in time happily engages in activities and his daughter comes to visit him and he doesn’t recognize his daughter anymore I could of course say; ‘Good afternoon, I am the doctor and I am going to give you an injection’. This was written down at one point by this person, but is it what he wants now?”(B)*

#### Law, due care criteria and the guidelines

Several participants considered the due care criteria as rather vague and open to differing interpretations. Particularly ‘unbearable suffering’ was seen as subjective and unclear. “*That’s the problem:.... it’s very difficult to establish to what extent the patient is suffering.” (E)* Participants also explained that recent guidelines are rather vague about the role of AEDs. One participant said: “*Patients expect they can give you their filled out form and that’s it, they think that is enough to receive euthanasia at the moment they want it*.” (G).

This opinion was not shared by all participants. For some, the law and guidelines were quite clear. A euthanasia request, according to one participant, can sometimes be accepted despite the patient’s mental incompetence or the patient’s lack of insights in the disease: *“You know, so, (the criterion of) being of sound mind and judgement should always be related to a particular situation. And if it is about euthanasia, I think that people with advanced dementia are still capable of expressing ‘I don’t want this, I’m suffering, I’m unhappy, I want out of this.‘ Here I don’t need a statement, I can tell from their misery. For example if someone is continuously banging on the door all day.”(H).*

Physician Assisted Suicide in the form of a lethal drink was mentioned several times as a better option than euthanasia through intravenous injection. It was seen as a more autonomous decision and expression of free will. “*To me this is very important, that people do it themselves, yes. Is this what this person wants? Then drink it yourself!”(C)*

### Expertise

#### Individual level

Most interviewees were not often confronted with euthanasia requests and procedures, despite the increasing frequency of euthanasia cases. Some participants stated that the more experience a doctor gains, the lower the emotional impact. “*Look, a GP will give euthanasia about once every two years- you see, that’s the frequency- well they will never get used to it. Every time, they have to climb the Himalaya again.”(C)*

#### Organisational level

According to some participants, the End-of-Life clinic also provides additional expertise and has more time to spend on euthanasia requests. But some participants had controversial views regarding the End-of-Life clinic, as they felt that performing euthanasia should not become the main practice of any physician, which often is the case for physicians working for this clinic. “*In 2017 the End-of-Life Clinic had 2700 requests, of which 850 were granted. And ehm, 8% of all the cases of euthanasia are accomplished by this club. This weird club. And ehm, we might see a development where euthanasia ends up with a small group of doctors who say ‘we’ll take care of it’. I don’t approve of it, but it might well happen.”(C)*

### Support and coping

#### Improving existing services

All participants acknowledged the importance of support for physicians and agreed the existing services could be improved, as well as the professionals’ awareness of these services. They all acknowledged that support and consultation provided by colleagues or by other medical professionals in the direct work environment, as well as peer review sessions, are helpful. Informal support from their own family was also mentioned several times. “*That is, for me at least, very important, that I can share this at home.”(G)* All elderly care physicians mentioned other forms of support they already used, such as a moral debate, consultation with a spiritual caregiver or a psychologist. One GP also mentioned the need for such support: “*Involve spiritual caregivers in the process. They are experienced in ethics and will ask questions which we as practitioners and diagnosticians often do not ask ourselves.”(F)*

#### Alternatives to euthanasia

Palliative care or allowing death from natural causes was mentioned by many participants as possible alternatives to euthanasia in particular cases**.** One participant stated that palliative sedation as a possible alternative to euthanasia should be further explored. *“In one case in our nursing home we chose palliative sedation because here there is a legal framework. And we may have to liberalize the guideline for palliative sedation [which states that it is only allowed for patients with a maximum remaining life expectancy of two weeks]. For dying within two weeks of dementia … that doesn’t seem realistic. And I’ve noticed that with palliative sedation there are fewer concerns so to speak.”(D).*

Some interviewees saw assisted suicide by a lay person as a more autonomous decision which should be legalized. [in the Netherlands, there is an organisation with trained consultants who provide information about self-euthanasia (the carefully considered realisation of the end of human life under own control); ‘the Einder’ [26]. As one participant stated: “*An organisation which assists with suicide. … When it became too much I referred to this club. There were some people who excelled in talking to people who were able to express their death wish. Sometimes this led to assisted suicide; then they were skirting the line of what is allowed.”(I)*

### Doctor’s emotions

Many participants mentioned that dealing with euthanasia requests is emotionally very intense. They described the euthanasia experience itself, and for most of them this concerned patients without dementia, as an unnatural experience. ***Negative emotions*** they experienced when dealing with euthanasia were feeling insecure, frustration, anger, moral distress, being judged by society and isolation. Some of them also felt stressed about the technical issues, such as administering the injection correctly, and about the possible legal consequences. “*Scared of the upheaval, the scandal, having killed someone, also the stigma and then the irreversibility of death. And that you killed someone …*. W*ell, that fear.”(C).*

***Positive emotions*** like heroism, being in control, satisfaction and relief were also mentioned. The following quote illustrates this with the legitimation of the physician’s power when having performed euthanasia: *“It’s wonderful … and also this feeling of power.*. *That’s, it’s a form of heroism. The feeling that I did something good, also for the profession.”(C)*

## Discussion

Using in-depth interviews, we explored the impact of euthanasia requests from PWD on Dutch GPs and elderly care physicians and their need for support. We interviewed eleven physicians with a wide variety of experience of euthanasia requests from PWD and differing attitudes towards euthanasia for this group of patients. Difficulties they experienced were related to timing, workload, pressure from relatives, society’s negative view of dementia in combination with the ‘right to die’ view, the interpretation of the law and AEDs, ethical considerations, and communication with PWD and relatives. The physicians we interviewed showed a larger variety of opinions than was found twelve years ago [9]. The adaptation of the KNMG guideline on this topic in 2015 might have contributed to these obvious changes. We also found that there is a lack of professional support.

Finding sufficient time to fully discuss a euthanasia request and, if applicable, perform euthanasia was considered problematic, in line with the findings of previous research [18, 27, 28]. Most participants in our study felt that the decision-making process with PWD takes more time. In our study the ‘culture of non-disclosure’ of the dementia diagnosis came forward. This is in line with an international systematic review study which showed that only 34% of GPs usually tell the PWD their diagnosis [29]. In addition, a Dutch study stated that physicians in general are not proactive when diagnosing dementia [30]. This has implications for PWD regarding participating in care planning and having sufficient time to discuss euthanasia requests.

Also dealing with an AED and evaluating the due care criteria in cases of mental incapacity was considered challenging. This confirms previous research also showing that physicians experience more difficulties evaluating the due care criteria in cases of dementia [4, 10, 28]. For instance, communication problems can make it very difficult to decide whether or not suffering is unbearable [28, 31].

Several participants experienced difficulties in dealing with pressure from relatives. This confirms findings of recent studies on dealing with euthanasia requests in general [22, 32, 33]. Also, most participants mentioned the negative view of Dutch society of dementia and the process of dying of PWD, which was phrased by Allan Kellehear as ‘shameful’ dying: ‘Dementia …. will deny most of us a good death or even a well-managed one.’ [34] Furthermore, some participants argued that the societal influences they experienced also result from our culture’s emphasis on autonomy and the view that euthanasia is synonymous with dignified dying. Kouwenhoven et al. also found this increased emphasis on the patient’s autonomous wish to be the primary basis for euthanasia [35]. Indeed, in a recent large national survey, keeping autonomy was in the top three of end-of-life aims of the Dutch population [36]. Society’s increasing focus on autonomy ‘as a right’ [6] has policy implications. A recent study, exploring the pressures experienced by Dutch GPs when dealing with euthanasia requests, called for more awareness in order to prevent physicians having to cross their own personal boundaries when dealing with AES requests [33]. Our participants considered the KNMG guideline to be vague and to provide too little guidance. The use of heuristics might be helpful in applying legal rules in daily practice when it concerns euthanasia [37].

The physicians in our study experienced both negative and positive emotions when confronted with a euthanasia request from a PWD; as has also been described regarding euthanasia requests in general [3, 38]. Participants mentioned feelings of insecurity, frustration, anger, moral distress, isolation and the feeling of being judged by society. Positive emotions mentioned by our participants were being in control, satisfaction, relief and even heroism. This feeling of heroism in the performance of euthanasia has not been described before, although a Dutch GP described ‘a feeling of power that ran through his body at the moment he performed his first euthanasia in a critical opinion article on euthanasia’. [39] Interestingly, one participant stated that as a physician’s experience with euthanasia increases, negative emotions tend to decrease and positive emotions to increase, which was considered undesirable. As, within the End-of-Life clinic, a limited number of physicians handle the euthanasia requests from PWD, meaning they have a much higher case load per physician, the emotional impact on this specific group of physicians needs further exploration.

Our participants mentioned using different forms of support when dealing with euthanasia requests from PWD. They mentioned existing services of support such as SCEN (support and consultation during euthanasia procedures*)*, consultation with palliative care teams, deliberation with other physicians and even their own family for emotional support. Some of these forms of support were also suggested in a study into the complexities in euthanasia perceived by Dutch physicians and relatives [40]. A striking finding of our study was that, although the participating elderly care physicians mentioned their use and appreciation of prospective ethical discussions or moral deliberation, this was hardly expressed by GPs. Such services are not easily available and accessible in primary care, hence they may be unaware of these options. As recently the Dutch government started to fund spiritual care in primary care, this might raise the awareness of GPs and give an opportunity to develop such support services.

### Strengths and limitations

In our study, both general practitioners and elderly care physicians, the groups of professionals who are most frequently confronted with euthanasia requests by PWD, were involved. However, this study also has limitations. Although we included physicians with a wide variety of opinions and experience on this topic and tried to provide a broad perspective, the majority of participants signed the critical statement about euthanasia in persons with advanced dementia [17]. Although saturation was reached, by interviewing just a small number of physicians on such a sensitive issue, we might have missed essential information.

## Conclusions

Euthanasia in dementia places an ethically and emotionally heavy burden on elderly care physicians and GPs in the Netherlands. The majority of the participants wish for more clarification of their professional guidelines. Existing useful support mechanisms, such as moral deliberation and support from chaplains are available for elderly care physicians. The awareness of, and access to, such support for GPs needs further exploration. Apart from the legal perspective, the interpretation of unbearable suffering and competence for people unable to express themselves needs more debate from psychological and ethical perspectives.

Quantitative insights into the problems and needs of physicians confronted with euthanasia requests from PWD are necessary. We suggest further research to identify ways of supporting physicians confronted with such requests.

## Data Availability

the transcriptions of the interviews, which are kept safely at Radboud University Medical center, are available from the corresponding author on reasonable request.

## Abbreviations

AED: Advance euthanasia directive
COREQ: Consolidated Criteria for Reporting Qualitative Studies
GP: General Practitioner
KNMG: Royal Dutch Medical Association
PWD: Persons with Dementia
